# A Perspective on Management of Limb Fractures in Obese Children: Is It Time for Dedicated Guidelines?

**DOI:** 10.3389/fped.2020.00207

**Published:** 2020-05-08

**Authors:** Fabrizio Donati, Pier Francesco Costici, Sergio De Salvatore, Aaron Burrofato, Enrico Micciulli, Aniello Maiese, Paola Santoro, Raffaele La Russa

**Affiliations:** ^1^Department of General Surgery, Orthopedic Institute, Bambino Gesù Children Hospital, Rome, Italy; ^2^Department of Orthopaedic and Trauma Surgery, Campus Bio-Medico University of Rome, Rome, Italy; ^3^Department of Anatomical, Histological, Forensic and Orthopaedic Sciences, Sapienza University of Rome, Rome, Italy

**Keywords:** childhood obesity, orthopedic, limb fractures, trauma surgery, clinical practice guidelines

## Abstract

Limb fractures are the most common injuries in pediatric orthopedics. Early and late complications are often not preventable, even when providing the best treatment; furthermore, these injuries are largely implicated in medico-legal claims. The development of evidence-based guidelines is one of the main goals of medical research. Approved guidelines for diagnosis, treatment, and follow up are fundamental to obtain the best results in medical practice. Guidelines in pediatric traumatology have been developed, even though specific conditions, like obesity, could influence their drafting. The cast and fixation systems usually applied in pediatric fractures provide a growth plate sparing, a satisfying reduction, and good stress resistance, mostly because of a lower bodyweight compared to adults. Several studies suggest that obesity influences the bone quality, the management, and the outcomes in cases of fracture. High body weight increases the risk of trauma, modifies fracture characteristics, and increases the risk of incomplete reduction. Fractures in obese children have a higher rate of complications, regardless of conservative or surgical treatment. In obese children, surgical treatment is often used more frequently than with non-obese children. Such considerations are valid both for lower and upper limb fractures. The aim of this paper is to discuss recent scientific literature and provide a perspective on the benefits of a dedicated approach in the management of obese children. Guideline updates could improve healthcare quality in a pediatric setting, and also reduce medico-legal implications.

## Introduction

Limb fractures are the most common injuries in pediatric orthopedics. Early and late complications are often not preventable, even when providing the best treatment; furthermore, these illnesses are often implicated in medico-legal claims. The development of evidence-based guidelines (EBG) is one of the main goals of medical research. Approved guidelines are fundamental to improve medical practice, especially in the management of specific diseases. The quality of guidelines strictly relies on the strength of its scientific evidence, which is often insufficient in pediatric orthopedic publications (1). A wide standardization of treatment is not always possible in medicine since specific conditions often require adjustments to the normal standard of care. One of these conditions is pediatric obesity (defined as a Body Mass Index, BMI, at or above the 95th percentile for children and teens of the same age and sex). Both obesity and polytrauma are major health problems. Despite rate of trauma admissions seemingly similar between obese and healthy patients (2), several studies found conflicting results regarding possible differences in injury patterns, severity, and outcomes (3, 4). Obese patients have different bone features (5) than healthy patients, and it could lead to different problems. Polytrauma is associated with systemic responses, and these may be modified in obese patients (6). Furthermore, obesity is associated with increases in the relative risk of deep venous thrombosis (DTV), pulmonary embolism (4), rate of decubitus ulcers (7), and other complications. It has been demonstrated that a higher rate of failure of non-surgical management of severe bone fractures is found with obese patients compared with normal-weighted children's fractures (8).

The aim of this paper is to highlight differences between fractured obese and non-obese pediatric patients and to provide a perspective on the benefits of a dedicated approach. All these differences between obese and non-obese pediatric patients emphasize the difficulty involved in creating dedicated EBG in pediatric trauma surgery, focused on both age and weight.

## Perspectives on Obese Children's Fractures

Several studies suggest that obesity could influence the natural history of pediatric limb fractures, modifying the standard treatment management (9, 10). A high body weight contributes to increasing the fracture risk and modifying bone proprieties, thus resulting in a less strong bone and consequently a higher risk of fracture. Obesity affects the bone quality and, consequently, the pattern of fractures. Dimitri et al. (11) have demonstrated that, despite an increased fracture risk, obese children have a higher bone mass; otherwise they have a lower total body and regional bone mass relative to their body size (12). Moreover, with the use of peripheral quantitative computed tomography in skeletal imaging, Cheung et al. (13) have shifted the focus in evaluating the changes in the architecture of trabeculae that result in a change in bone quality and strength. The results show that an increased body size is not related to increased bone strength. Trabecular bone in obese children appears to undergo a trabecular reorganization. In fact, the increasing of trabecula with a reduction in trabecular spacing confers a theorical structural advantage (14, 15). In addition to altered mechanical proprieties, there are differences in the onset of puberty due to the vitamin D deficiency and the direct effects of adipokines, such as leptin (16).

A specific correlation between BMI and site of fracture has been demonstrated. A recent study (17) conducted on 449 young patients showed an increased rate of upper and lower limb fractures in obese girls and a higher rate of lower limb fractures in obese boys compared to the normal weighted group. Ryan et al. (18) reported a higher rate of forearm fractures from ground-level falls in children with increased weight status. Greater body mass index is associated with increased odds of lower extremity fractures and pain issues, such as musculoskeletal pain complaints, sprains, and dislocation (19). Kessler et al. (20) examined the association between body weight and lower extremity fracture site and found that obesity was associated with increased odds of fracture of the foot, ankle, leg, and knee, particularly in patients between 6 and 11 years old. This study also showed there were no associations between obesity and fracture risk for the femur or hip.

Obesity causes several complications in the management of fractures. Hirsch et al. (21) examined the rates of oxygen desaturation during procedural sedation for manipulative reduction of long bone fractures. They found obese patients had greater desaturation rates (9.9% vs. 5.4%) than the healthy population. Pediatric obesity is also associated with an increased death rate following severe fractures (22).

### Upper Extremity Fractures

**Figure 1:**
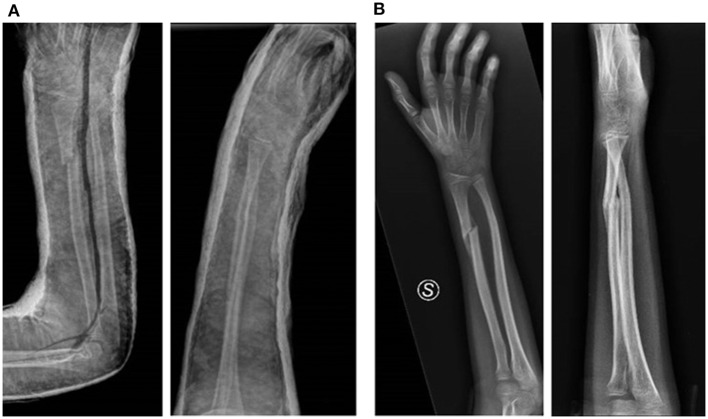
Left forearm fracture in 11-year-old obese patient, treated conservatively **(A)**. A 1-month X-ray follow-up shows a significant loss of reduction was evident, with improved angulation of the fragments **(B)**.

Pediatric forearm fractures commonly involve the radius and ulna. They are the most frequent pediatric fractures and several studies (7) have demonstrated a higher incidence of extremity fractures in obese patients (55% vs. 40%). The same study also noted a greater rate of surgical treatment in obese pediatric patients than in non-obese ones. The study of Pomerantz et al. (23) found no differences in the incidences of upper extremity fractures in obese and healthy patients (36.3 vs. 36.3%); however, they found a difference regarding the lower extremity (obese patients had a greater rate at 42% vs. 30%). Auer et al. (24) reviewed 157 distal radius fractures and highlighted that obese patients were more likely to require a second reduction (28% vs. 12%). Regarding the conservative treatment, studies by Okoroafor et al. (8) and DeFrancesco et al. (25) showed that obese patients with diaphyseal forearm fractures (both ulna and radius) were more likely to have an incomplete reduction after cast treatment (Figure 1).

Concerning elbow fractures, in a study of 992 patients, Fornari et al. (26) found that lateral condyle fractures were more common in obese patients than non-obese patients (37% vs. 19%). In the same study, they found that the percentage of obese patients affected by a supracondylar humerus fracture was similar to normal weighted ones.

The loss of reduction is also a problem that affects elbow fractures. Chang et al. (27) showed, in a study of 107 patients affected by type 3 supracondylar humerus fractures, a correlation between obesity and a higher risk to develop postoperative loss of reduction and varus alignment. Sangkomkamhang et al. (28) studied 256 patients with supracondylar humerus fractures treated with open and closed reduction and found loss of reduction in 14.8%. Even though the result of the study was influenced by poor surgical technique, they found an association between BMI > 25 kg/m^2^ and increased risk of loss of reduction. Li et al. (29) described a correlation between age, obesity, and need of open reduction in patients affected by supracondylar humerus fractures. In fact, they found that obese patients between 8 and 12 years old were over four times more likely to require open reduction compared with healthy patients. There were no differences in patients between 2 and 7 years old (Figure 1).

### Lower Extremity Fractures

Being overweight increases the odds of sustaining injury to lower extremities (30); a review on 3,232 cases of children involved in motor vehicle collisions found that the obese population had an increased risk of fractures compared to a healthy one. Furthermore, the prognostic study conducted by Gilbert et al. (31) reported that obese patients were twice as likely to have fractures involving the physis compared to non-obese patients in obese patients (with a relative risk of RR 2.2, 95% confidence interval, 1.25–3.89). Authors analyzed patients of 2–14 years of age who had been admitted following high-energy trauma alerts. Three hundred and ninety seven patients with femur and/or tibia fractures were identified. Typical causes of the trauma included motor vehicle crashes, falls from a height, and being struck by a vehicle. Weight, height (when available), age, and fracture location and method of treatment were recorded. Results showed that obese patients had a higher proportion of epiphyseal fractures at all locations, and the differences reached statistical significance for the proximal femur. The increased propensity for physeal fracture in obese patients is likely to be multi-factorial including physiologic and mechanical influences. Fat may have endocrine effects on bone through obesity-related pathways; obesity may also place chronic excessive stress on the bones and cartilage as well as the supporting ligaments and tendons. Kessler et al. (20) performed a huge cohort study (almost 1 million patients), dividing patients by age and weight, and found a linear correlation between a patient's weight and risk of fracture in the lower limbs. Extremely obese children (BMI > 35 kg/m^2^) had 1.4 odds of fracture, while moderately obese (BMI 30–35 kg/m^2^) had 1.23 odds of sustaining a fracture compared to the healthy population. Leet et al. (9) demonstrated that obese children with lower limb fractures have an increased rate of postoperative complications compared with non-obese ones. They performed an epidemiologic study of 356 patients and reported that obese patients with femur and tibia fractures have more severe injuries, predisposing them to greater morbidity and mortality.

Concerning the risk of loss of reduction when treating femur fractures with elastic nails, there are conflicting results. Several studies found a correlation between obese patients and an increased risk of loss of reduction and malunion (32, 33). Conversely, a study made by Nielsen et al. (34) reported no association between BMI and malunion. Such results could be influenced by the choice of different surgical treatment in patients with a weight higher than 45 kilograms where other more stable and invasive fixation systems are indicated.

Tibia fractures have not demonstrated strict weight limitations when using elastic nails. Goodbody et al. (35) reviewed 95 adolescents with tibia fractures without finding a significant difference in the rate of malunion or healing time between obese and non-obese patients.

No correlations between obesity and complications relating to external fixation of lower extremities were found in the study by Fedorak et al. (36).

Kessler et al. (20) found the strongest correlation between obesity and fractures of the foot and ankle, even though few studies have been published about these fractures in overweight children.

## Discussion

The prevalence of childhood obesity increases every year; in the US the prevalence between 2011 and 2014 was 17.0% (37) and extreme obesity was 5.8%, hence it is necessary to consider the obese population in the guidelines making process because they represent a wide population.

In 1990, Field and Lohr (38) described clinical practice guidelines as “systematically developed statements to assist practitioners and patient decisions about appropriate health care for specific circumstances.” Guidelines are intended to offer a brief instruction on how to provide healthcare services which are up-to-date and in accordance with the latest progress, assisting the clinicians in the decision-making process. An appropriate interpretation and application of guidelines can generate better clinical care and a safer medicolegal strategy. Clinical practice guidelines are considered as one of the most effective tools for the promotion of evidence-based medicine (EBM).

Evidence-based medicine (EBM) has been quoted as “the integration of best research evidence with our clinical expertise and our patient's unique values and circumstances.” (39). The application of evidence-based medicine in clinical practice is an approach to patient care that emphasizes knowledge of the best clinical evidence available for making the correct diagnosis and treatments. There are three fundamental principles of EBM. The first is that the optimal clinical decision requires awareness of the best and last available evidences. The second is that the EBM provides guidance to decide whether evidence is reliable. The last is that the evidence alone is never sufficient to make clinical decisions. These reasons make the developing of EBM guidelines a difficult and time-consuming process (40).

The World Health Organization has stated that governments must take responsibility for their healthcare systems and has proposed an active involvement in the quality of services provided (41).

In the Italian perspective, the government agenda for healthcare included the enactment of a law (law n° 24, 2017) containing provisions relating to the institution of a National Guidelines System. This law encourages healthcare providers to use clinical practice guidelines included in the official lists, to ensure uniformity and consistency of healthcare throughout the country. The Italian Ministry of Health promotes healthcare safety as a relevant part of the “right to health.”

In pediatric orthopedics there are few high-quality level publications, making the guidelines creation process difficult. In fact, the majority of studies available are levels III or IV of evidence (42). Moreover, the pediatric population needs a very long time follow up to asses a good long-term result. It is important to create dedicated committees to produce pediatric guidelines. In fact, the American Academy of Orthopedic Surgeons (AAOS) has a dedicated committee that produces clinical practice guidelines on a variety of pediatric issues (43) and it should be one of the most important goals of all the national scientific societies. However, few studies are focused on other aspects of patients rather than age and fracture site or type. Weight should be considered as a separate criterion in pediatric fracture guidelines, and not only in lower limb fractures.

It is mandatory to create a standard classification of pediatric fractures to make specific guidelines and to elaborate on the decision algorithm when choosing the fixation system. Various authors and scientific societies tried to do it, but probably the most accurate classification was provided by AO society (44). Another important classification was made by Salter-Harris and is focused on growth plate injuries (45). The cast and fixation systems usually applied in pediatric fractures provide a growth plate sparing, a satisfying reduction, and good stress resistance, mostly because of a lower bodyweight compared to adults. Fractures in obese children have a higher rate of complications independently from conservative or surgical treatment. Surgical indications are more common than in normal weighted children and are generally more invasive. Such considerations are valid both for lower and upper limb fractures.

The rising rate of obesity is one of the most urgent challenges to public health; the number of obese children (aged 0 to 5 years) increased to 41 million globally in 2016. Childhood obesity is associated with a range of serious health complications and an increased risk of premature onset of illnesses such as diabetes and heart diseases. Obesity increases the risk of malpractice litigation, and obesity-related claims have dangerously increased in the last decade.

Obese children have genetic, hormonal, and clinical differences compared to healthy patients. Obesity is associated with altered metabolic patterns and changes in bone composition (5, 10, 13). The altered bone structure and bone mass accrual are related to higher fracture rates, and these altered bones are more likely to sustain growth plate fractures in the limbs. A correlation between a high BMI (>30 kg/m^2^) and increased risk in limb fractures has been proven. It has also been proven that obese patients have a site-specific weakness of bone forearms exposing them to high risk of re-fractures (5). Moreover, they have an increased risk for complications (6), respiratory diseases that force prolonged ventilation (46), and an increased rate of mortality (9). Obese children have a higher risk of loss of reduction of forearm fractures treated both by casting and open reduction (3). Obesity is also related to an increased rate of open reduction and internal fixation of limb fractures (10).

## Conclusion

According to our perspective, standard guidelines are not always applicable in obese children, who often require specific care strategies. As a consequence, we highlight the necessity of periodic guideline updating based on literature review, allowing a patient-specific medical approach. In particular, the development of dedicated guidelines for the management of limb fractures in obese children could avoid several legal implications.

## Author Contributions

FD, PC, AB, and EM designed the study and wrote the manuscript. SD and PS provided data from literature. RL and AM revised the manuscript.

## Conflict of Interest

The authors declare that the research was conducted in the absence of any commercial or financial relationships that could be construed as a potential conflict of interest. The handling Editor declared a shared affiliation, though no other collaboration, with several of the authors [AM, PS, RL].
